# Lactate Activates HIF-1 in Oxidative but Not in Warburg-Phenotype Human Tumor Cells

**DOI:** 10.1371/journal.pone.0046571

**Published:** 2012-10-17

**Authors:** Christophe J. De Saedeleer, Tamara Copetti, Paolo E. Porporato, Julien Verrax, Olivier Feron, Pierre Sonveaux

**Affiliations:** Pole of Pharmacology, Institut de Recherches Expérimentales et Cliniques (IREC), Université catholique de Louvain (UCL), Brussels, Belgium; University of Texas Health Science Center at San Antonio, United States of America

## Abstract

Cancer can be envisioned as a metabolic disease driven by pressure selection and intercellular cooperativeness. Together with anaerobic glycolysis, the Warburg effect, formally corresponding to uncoupling glycolysis from oxidative phosphorylation, directly participates in cancer aggressiveness, supporting both tumor progression and dissemination. The transcription factor hypoxia-inducible factor-1 (HIF-1) is a key contributor to glycolysis. It stimulates the expression of glycolytic transporters and enzymes supporting high rate of glycolysis. In this study, we addressed the reverse possibility of a metabolic control of HIF-1 in tumor cells. We report that lactate, the end-product of glycolysis, inhibits prolylhydroxylase 2 activity and activates HIF-1 in normoxic oxidative tumor cells but not in Warburg-phenotype tumor cells which also expressed lower basal levels of HIF-1α. These data were confirmed using genotypically matched oxidative and mitochondria-depleted glycolytic tumor cells as well as several different wild-type human tumor cell lines of either metabolic phenotype. Lactate activates HIF-1 and triggers tumor angiogenesis and tumor growth *in vivo*, an activity that we found to be under the specific upstream control of the lactate transporter monocarboxylate transporter 1 (MCT1) expressed in tumor cells. Because MCT1 also gates lactate-fueled tumor cell respiration and mediates pro-angiogenic lactate signaling in endothelial cells, MCT1 inhibition is confirmed as an attractive anticancer strategy in which a single drug may target multiple tumor-promoting pathways.

## Introduction

Cancer is a disease striving to match tumor cell ATP production and demand and to fulfill the biosynthetic needs for proliferation in a microenvironment heterogeneously providing oxygen and nutrients [1], [2]. Full glucose oxidation to water and CO_2_ is an efficient mode of energy production generating up to 38 molecules of ATP per molecule of glucose. It requires a functional coupling between glycolysis and oxidative phosphorylation (OXPHOS), and oxygen as the final electron acceptor of the respiratory chain. However, hypoxia is impacting glucose utilization in most solid tumors [1]. Low pO_2_ indeed fosters a glycolytic switch, formally corresponding to uncoupling glycolysis from OXPHOS, initially imposed by alleviation of the negative feed-back exerted by energy metabolites on the glycolytic flux (the Pasteur Effect) [3]. Compared with full glucose oxidation, glycolysis alone is a poor energy provider, yielding only 2 molecules of ATP per molecule of glucose. Therefore, if hypoxia persists or if the pO_2_ continuously fluctuates, several oxygen-, nutrient- and energy-sensing systems cooperate to further increase the glycolytic rate [2], thus resulting in an important demand for glucose and elevated lactate production. Importantly, glycolysis also offers the necessary plasticity needed to fuel the biosynthetic pathways supporting cell proliferation [2]. To fulfill their proliferative agenda, tumor cells (TCs) therefore evolve constitutive glycolysis, a metabolic phenotype known as the Warburg effect [4]. Little is known about the genetic and epigenetic changes driving the Warburg phenotype. Although mutations in mitochondrial enzymes have been identified in several cancer cell lines [5]–[7], increasing pieces of evidence indicate that the Warburg effect can most often be reverted pharmacologically [8]–[10].

Core to the machinery supporting the glycolytic switch is activation of hypoxia-inducible factor-1 (HIF-1), a transcription factor interfacing hypoxia and the upregulation of genes encoding most glycolytic transporters and enzymes, including enzymes insensitive to or bypassing the Pasteur Effect [2], [11], [12]. HIF-1 is a dimeric αβ complex. Its activation control essentially depends on the posttranslational stabilization of the HIF-1α subunit, whereas HIF-1β/ARNT is constitutively nuclear [13]. With a low K_m_ for oxygen [14], Fe(II)- and 2-oxoglutarate-dependent dioxygenase prolylhydroxylase 2 (PHD2) is considered as the oxygen sensor of the system [15]. Under normoxia, PHD2 transfers 2 hydroxyl groups onto proline residues 402 and 564 (human sequence) of the oxygen-dependent domain (ODD) of HIF-1α, thereby targeting this subunit for poly-ubiquitylation by the Von Hippel-Lindau (VHL) protein complex followed by proteasomal degradation [16]. Conversely, prolylhydroxylations are blunted under hypoxia, allowing the translocation of significant amounts of the HIF-1α protein into the cell nucleus where it binds to HIF-1β and other cofactors to form a transcriptionally active multiproteic complex. Besides this canonical hypoxia-driven pathway, several alternative routes allow normoxic HIF-1 activation, either because they interfere with redox cycling of the iron prosthetic group of PHD2 (as it is the case with nitric oxide and reactive oxygen species) [17], or because they compete with 2-oxoglutarate for the PHD2 reaction (as exemplified with pyruvate and dimethyloxalylglycine) [18]–[21].

Functional competition between pyruvate (largely originating from glycolysis) and 2-oxoglutarate (formed in the cytosol from the cataplerotic intermediate citrate) could provide a molecular coupling between a glycolytic metabolism and HIF-1 activation, should glycolysis be induced by hypoxia or be constitutive as it is the case in Warburg TCs. Accordingly, we recently reported that this precise mechanism accounts for normoxic HIF-1 activation in nonmalignant endothelial cells exposed to exogenous lactate at concentrations commonly found in tumors [22]. In these cells, lactate is oxidized into pyruvate by lactate dehydrogenase-1 (LDH1) and thereby supports the competitive inhibition of PHD2 by pyruvate accounting for HIF-1 activation under normoxia. This response to lactate appears to be more complex in tumor cells. Indeed, although Lu *et al.* [18] reported HIF-1α protein stabilization by pyruvate (1–3 mM) in several lines of normoxic TCs, the level of lactate-induced HIF-1α protein stabilization was inconsistent among cell lines. Others [23] further failed to show any PHD2 inactivation by pyruvate. Thus, whether intrinsic characteristics of TCs influence the normoxic activation of HIF-1 by lactate remains an open question.

Constitutive HIF-1 activity has been proposed to be associated with aerobic glycolysis in the particular case of VHL-deficient renal cell carcinomas [24]. We therefore reasoned that the metabolic status of TCs could impact the response to lactate and to hypoxia. This hypothesis was tested in conditions wherein well characterized oxidative and Warburg-phenotype human TCs were exposed to exogenous lactate at concentrations commonly found in human tumors (1–40 mM) [25]. We report that under normoxia lactate activates HIF-1 in oxidative but not in Warburg-phenotype TCs.

## Results

### Under normoxia, lactate activates HIF-1 in oxidative but not in Warburg-phenotype tumor cells

**Figure 1:**
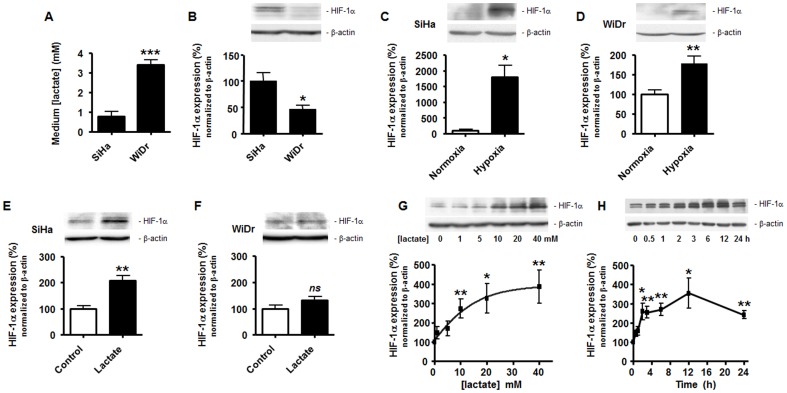
Lactate induces normoxic HIF-1α protein stabilization in oxidative tumor cells, not in Warburg tumor cells. (A) Lactate release in the supernatant of SiHa and WiDr TCs was measured using a CMA600 enzymatic analyzer after 24-h of culture in fresh medium. ***p = 0.0002; *n* = 4. (B–G) HIF-1α and β-actin protein expression was detected using Western blotting in the lysates of oxidative SiHa or Warburg WiDr TCs. The upper panels show representative experiments and the graphs HIF-1α protein expression normalized to β-actin levels. (B) SiHa and WiDr cells were untreated to detect basal HIF-1α protein expression. *p = 0.0414; *n* = 3–4. (C) SiHa TCs were cultured during 24-h under hypoxia (1% O_2_) or not. *p = 0.011; *n* = 3. (D) As in (C) but with WiDr TCs. **p = 0.0057; *n* = 5. (E) SiHa TCs were cultured during 24-h in the presence of 10 mM lactate or not. **p = 0.0029; *n* = 4. (F) As in (E) but with WiDr TCs. *ns*, *p* = 0.1449; *n* = 8. (G) SiHa TCs were exposed to increasing doses of lactate during 24-h. *p<0.05, **p<0.01 *versus* 0 mM lactate condition; *n* = 9–11. (H) SiHa TCs were exposed to 10 mM lactate during increasing periods of time. *p<0.05, **p<0.01 *versus* time 0; *n* = 3.

This study was aimed to test whether the metabolic status of TCs influences HIF-1 activation by relevant tumor microenvironmental stimuli. The hypothesis was initially addressed using the well characterized human TC lines SiHa (a human cervix squamous cell carcinoma cell line with oxidative metabolic activities) and WiDr (a human colorectal adenocarcinoma cell line performing aerobic glycolysis, i.e., of the Warburg phenotype) [26]. Metabolic profiles were confirmed *in vitro* by showing that WiDr TCs released about 3.5-fold more lactate that SiHa TCs over a 24-h period under normoxia (Figure 1A). We first found that WiDr TCs express significantly less basal levels of HIF-1α protein compared to SiHa TCs (Figure 1B). Canonical HIF-1 activation in both cell types was induced using hypoxia (1% O_2_, 24-h), with a much stronger stabilization of the HIF-1α protein in oxidative SiHa TCs (Figure 1C) than in Warburg-phenotype WiDr TCs (Figure 1D). A ∼18-fold HIF-1α induction was detected in SiHa TCs, which corresponded to ∼10 times the HIF-1α induction that was measured in WiDr TCs.

Lactate-induced HIF-1α protein stabilization was evaluated by exposing normoxic TCs during 24-h to 10 mM lactate, a concentration corresponding to the average level of lactate detected in human tumors [27]. In normoxic SiHa TCs, lactate (Figure 1E) similar to pyruvate (Figure S1A) induced a significant increase in HIF-1α protein expression, whereas normoxic WiDr TCs did not respond to lactate (Figure 1F). Experimental variability was found using a same cell line at different passages, with the level of HIF-1α induction by lactate ranging from +30% (Figure S1) to +110% (Figure 1E). In SiHa TCs, exogenous lactate triggered a concentration-dependent increase in HIF-1α protein expression, which was statistically significant within the range of 10 to 40 mM (Figure 1G). The latter concentration corresponds to the highest level of lactate ever detected in human tumors [25]. The response was also time-dependent with a plateau corresponding to a ∼2.5-fold increase in HIF-1α protein expression reached at 3 h after incubation with 10 mM lactate and maintained up to 24 hours following treatment (Figure 1H). Statistical variability between samples was the lowest at 24-h.

**Figure 2:**
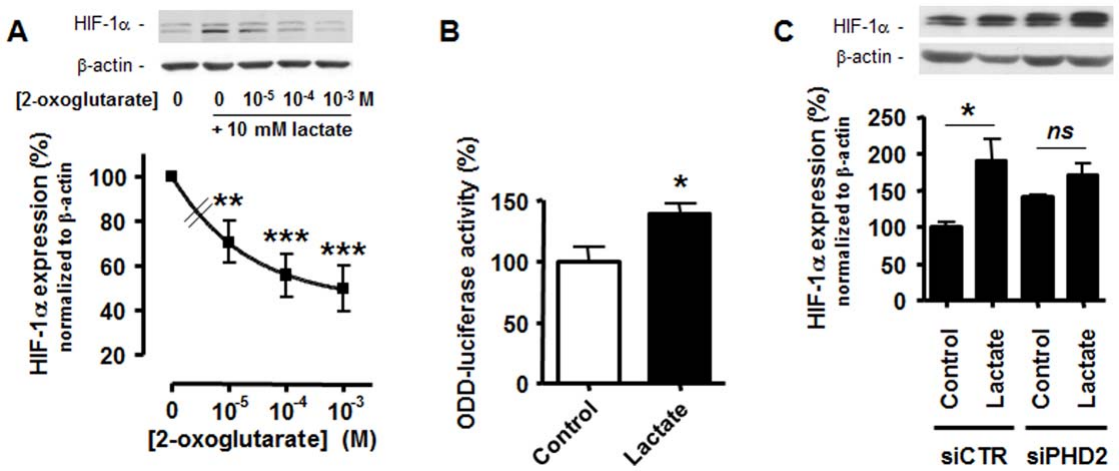
Lactate inhibits PHD2 activity in oxidative tumor cells. (A) HIF-1α and β-actin protein expression was detected using Western blotting in the lysates of SiHa TCs incubated during 24-h with 10 mM lactate or not and increasing doses of 2-oxoglutarate. The upper panels show representative experiments and the graph HIF-1α protein expression normalized to β-actin levels. Data are expressed as % of lactate induction. **p<0.01, ***p<0.005 *versus* 10 mM lactate without 2-oxoglutarate; *n* = 8. (B) ODD-driven luciferase activity was measured in SiHa TCs treated during 24-h with 10 mM lactate or not. *p = 0.0206; *n* = 6. (C) HIF-1α and β-actin protein expression was detected using Western blotting in the lysates of SiHa TCs transfected with a specific siRNA against PHD2 (siPHD2) or with a control siRNA (siCTR) and incubated during 24-h with 10 mM lactate or not. The upper panels show representative experiments and the graph HIF-1α protein expression normalized to β-actin levels. *ns*, *p*>0.05, *p<0.05; *n* = 3.

Because the lactate incubation of SiHa TCs induced no change in *HIF-1α* mRNA expression over time (Figure S1B), we tested whether lactate supported a posttranslational stabilization of HIF-1α in these cells. Based on previous data in tumor and nonmalignant cells [18], [19], [21], [22], we focused on the PHD reaction and competed exogenous lactate with the PHD substrate 2-oxoglutarate. We found that 2-oxoglutarate dose-dependently inhibited lactate-induced HIF-1α protein expression in normoxic SiHa TCs (Figure 2A). To directly measure PHD activity, we used an ODD-luciferase reporter in which the oxygen-dependent domain of HIF-1 posttranslationally controls luciferase protein expression [17]. Renilla luciferase served for transfection normalization. We found that 10 mM lactate significantly inhibited PHD activity as it increased the luciferase signal detected in SiHa TCs 24-h after treatment (Figure 2B). Conversely, while silencing PHD2 with a specific siRNA (see Figure S2A for target extinction) induced an expected increase in HIF-1α protein expression (Figure 2C), it also abrogated lactate-induced HIF-1α protein stabilization, thus confirming that PHD2 inhibition participates in the stimulation of HIF-1α by lactate.

**Figure 3:**
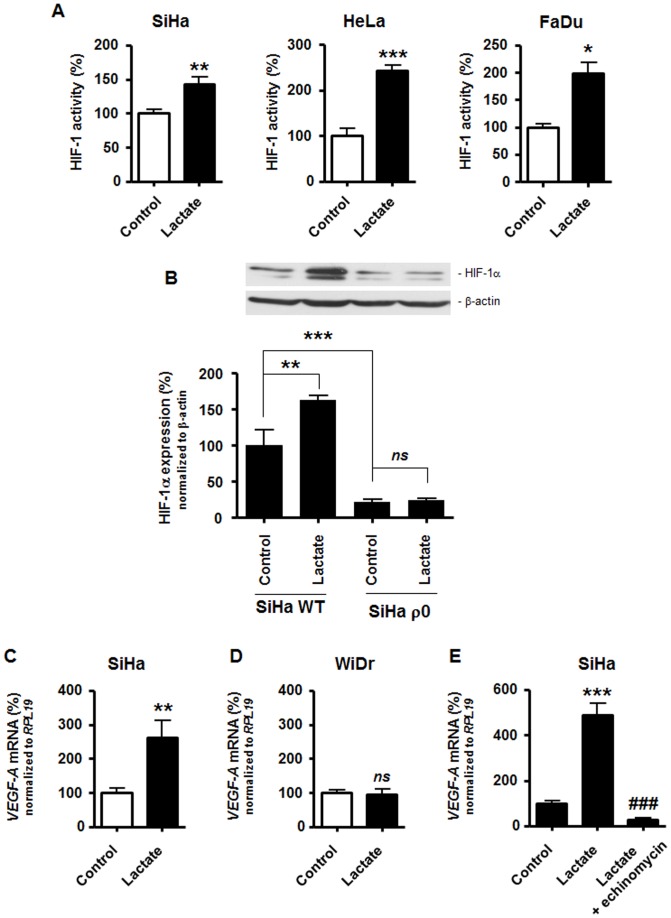
Lactate activates HIF-1 in normoxic oxidative tumor cells. (A) HIF-1 activity was quantified using a dual reporter luciferase assay in oxidative SiHa (left panel, *n* = 6–8), HeLa (middle panel, *n* = 3–4), and FaDu cancer cells (left panel, *n* = 3–4). All cells were cultured during 24-h in fresh medium containing 10 mM lactate or not (control). *p<0.05, **p<0.01, ***p<0.005. (B) HIF-1α and β-actin protein expression was detected using Western blotting in the lysates of wild-type (WT, *n* = 3) or mitochondria-depleted (ρ0, *n* = 5–6) SiHa TCs cultured during 24-h in the presence of 10 mM lactate or not. The upper panels show representative experiments and the graphs HIF-1α protein expression normalized to β-actin levels. *ns*, *p*>0.05, **p<0.01, ***p<0.005. (C–E) The level of *VEGF-A* transcript was determined using RT-qPCR in TC lysates. (C) SiHa TCs were exposed to 10 mM lactate or not during 24-h. **p = 0.009; *n* = 6. (D) As in (C) but with WiDr TCs. *ns*, *p*>0.05; *n* = 3. (E) SiHa TCs were cultured during 24-h in the presence of 10 mM lactate, 10 mM lactate+10 nM echinomycin (an inhibitor of the transcriptional activity of HIF-1), or none of these drugs (control). ***p<0.005 *versus* control; ^###^ *p*<0.005 *versus* lactate condition; *n* = 3–4.

Activation of HIF-1 by lactate under normoxia was verified in several oxidative TC lines. The oxidative nature of the selected cells has been demonstrated in previous publications based on either thorough metabolic profiling (SiHa human squamous cervix cancer cells [26]), addiction to glutamine rather than glucose (HeLa human epithelial cervix cancer cells [28]), or a switch from oxidative to glycolytic ATP production upon mitochondrial inhibition (FaDu human squamous pharynx cancer cells [29]). To determine HIF-1 activity, we used a dual luciferase reporter assay in which firefly luciferase was under the transcriptional control of HIF-1 [16]. Renilla luciferase served for transfection normalization. Lactate (10 mM, 24-h) induced significant HIF-1 activation in normoxic SiHa, HeLa, and FaDu TCs (Figure 3A). Although significant induction was observed for all the oxidative TC lines, the level of induction varied among them. Lowest and highest HIF-1 activation levels in response to lactate were seen in SiHa and HeLa TCs, respectively. This hierarchy was conserved when analyzing hypoxia-induced HIF-1 activation (Figure S3). In contrast with oxidative TCs, we detected no activation of HIF-1 by lactate in Warburg-phenotype TCs; these results are detailed in the next paragraph. To exclude metabolism-independent influences inherent to different genotypes, we further compared the response to lactate of wild-type oxidative *versus* mitochondria-depleted (ρ0) glycolytic SiHa TCs (see reference [26] for the metabolic characterization of these cells). The lactate-induced HIF-1α protein stabilization observed in wild-type TCs was totally absent in isogenic ρ0 TCs which also expressed lower basal levels of HIF-1α (Figure 3B). It confirms that aerobic glycolysis *per se* confers resistance to lactate signaling. To fully validate differential HIF-1 activation by lactate in oxidative *versus* Warburg-phenotype TCs, we checked the transcription of *vascular endothelial growth factor-A* (*VEGF-A*), a well-known HIF-1-target gene [30] reported to be inducible by lactate [18]. Using quantitative RT-PCR (RT-qPCR), a ∼2.6-fold increase in *VEGF-A* transcription was detected 24-h after the treatment of oxidative SiHa TCs with 10 mM lactate (Figure 3C), while *VEGF-A* expression was not influenced by lactate exposure in WiDr glycolytic TCs (Figure 3D). The involvement of HIF-1 in lactate-induced *VEGF-A* transcription in SiHa TCs was verified using echinomycin, an inhibitor of the transcriptional activity of HIF-1 [31]. As expected, RT-qPCR performed on an independent set of samples showed total inhibition of lactate-induced *VEGF-A* mRNA expression in the presence of echinomycin (Figure 3E).

### MCT1 inhibition blocks lactate-induced HIF-1 activation in oxidative tumor cells

**Figure 4:**
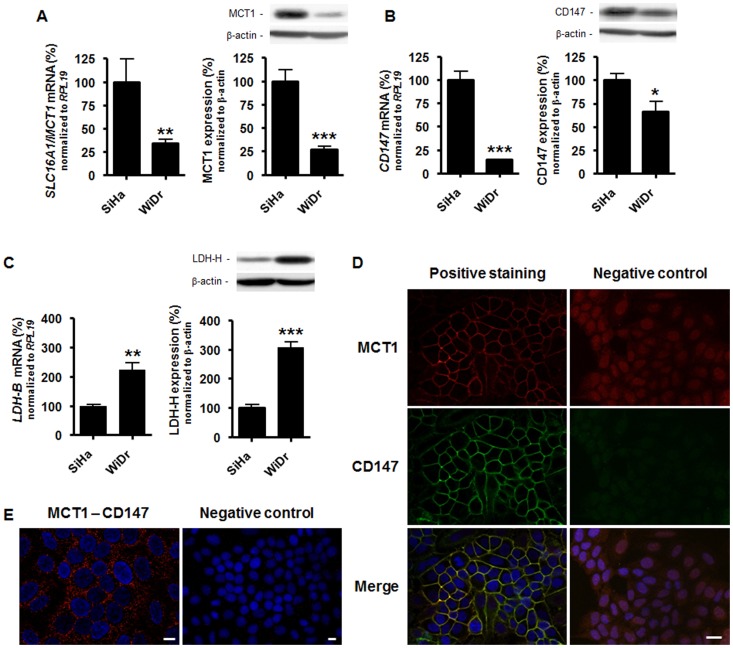
MCT1 and CD147 interact at the plasma membrane of oxidative tumor cells. (A) The left graph shows basal *SLC16A1*/MCT1 mRNA expression normalized to *RPL19* mRNA expression detected in SiHa and WiDr TCs using RT-qPCR. **p = 0.0062; *n* = 5–8. On the right, MCT1 and β-actin were detected using Western blotting (WB) in cell lysates. The upper panels show a representative experiment and the graph HIF-1α protein expression normalized to β-actin levels. ***p = 0.0004; *n* = 5. (B) as in (A) but detecting CD147 instead of MCT1. RT-qPCR: ***p<0.0001; *n* = 7. WB: *p = 0.0237; *n* = 6. (C) as in (A) but detecting *LDH-B* and LDH-H instead of MCT1. RT-qPCR: **p = 0.008; *n* = 3. WB: ***p<0.001; *n* = 6. (D) MCT1 (red, left upper panel) and CD147 (green, left medium panel) were detected using immunocytofluorimetry in SiHa TCs. The lower left panel is a merged picture in which cell nuclei have been stained in blue (Hoechst 33342). Right panels show control experiments in which primary antibodies were omitted. Pictures are representative of *n* = 4. Bar = 20 µm. (E) The *in situ* interaction between MCT1 and CD147 was verified in SiHa TCs using a proximity ligation assay. MCT1-CD147 interaction is identified with a red staining and cell nuclei are in blue (Hoechst 33342) in the left panel. The right panel shows control experiments in which the primary antibody against CD147 was omitted. Pictures are representative of *n* = 6. Bars = 10 µm.

Because lactate as an anion requires transporters to efficiently cross cell membranes, we next tested whether hypoxia mimicry by lactate could be blocked at the transporter level. Four monocarboxylate-proton symporters, MCT1 to MCT4, can transport lactate [32], [33]. Among these, we found that *SLC16A1*/MCT1 is the main transcript expressed by SiHa TCs (Figure S4A). *SLC16A3*/MCT4 mRNA was expressed at a ∼5-fold lower level than *SLC16A1*/MCT1 mRNA. *SLC16A7*/MCT2 and *SLC16A8*/MCT3 were barely detectable. MCT1 has now been confirmed to be the main progenitor of lactate uptake by TCs [26], [34], whereas MCT4 has a low affinity for lactate but a high turnover rate and primarily conveys lactate export from glycolytic TCs [35]–[37]. We therefore focused on MCT1. Comparisons showed that oxidative SiHa TCs express consistently higher levels of MCT1 than glycolytic WiDr TCs, which was observed at both mRNA and protein levels (Figure 4A). The membrane targeting, function and stability of MCT1 depends on its interaction with chaperone protein CD147/basigin [38], [39]. Accordingly, we found increased *CD147* transcript levels and higher CD147 protein expression in SiHa *versus* WiDr TCs (Figure 4B), thus suggesting that oxidative TCs are better equipped to import lactate than Warburg-phenotype TCs. Lactate-induced HIF-1 activation also depends on the oxidation of lactate to pyruvate, a process catalyzed by LDH1 [21], a tetrameric enzyme composed of 4 LDH-H subunits encoded by the *LDH-B* gene [2]. Although both SiHa and WiDr TCs consistently expressed LDH-H, we detected higher *LDH-B* mRNA and LDH-H protein levels in WiDr *versus* SiHa TCs (Figure 4C). In SiHa TCs, we further documented a plasma membrane colocalization of MCT1 and CD147 using immunofluorescent staining (Figure 4D). This interaction was verified using a proximity ligation assay (PLA) [40]. MCT1-CD147 complexes were detected in the cytosol but also notably at the plasma membrane of SiHa TCs (Figure 4E). Omission of the primary antibody against CD147 was used as a negative control resulting in total loss of the PLA signal.

**Figure 5:**
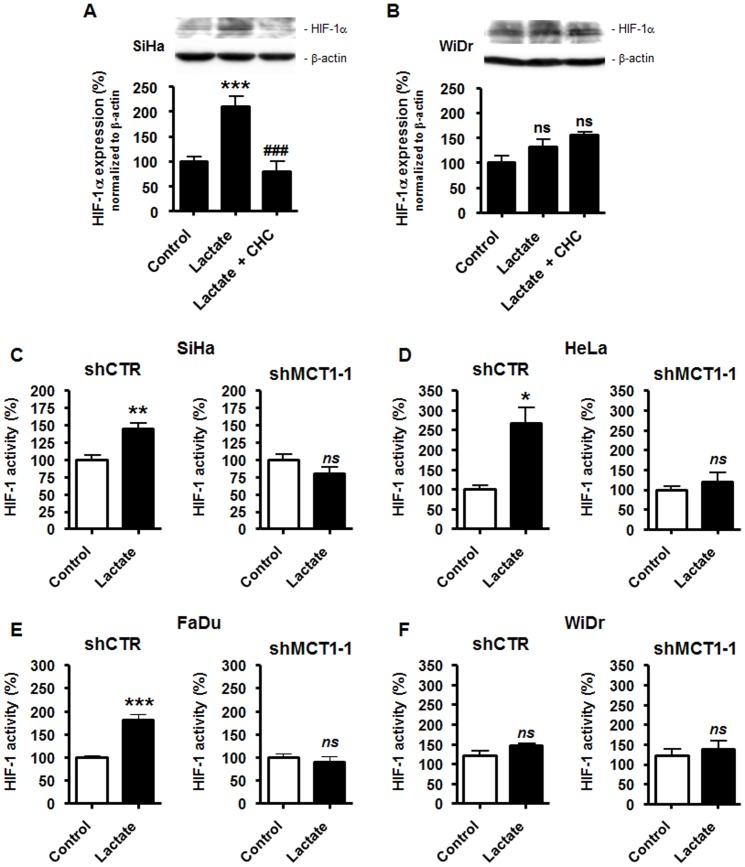
Targeting MCT1 inhibits lactate-induced but not basal HIF-1 activity in tumor cells. (A) SiHa TCs were cultured during 24-h in fresh medium containing 10 mM lactate, lactate +5 mM α-cyano-4-hydroxycinnamate (CHC), or none of the drugs. HIF-1α and β-actin were detected using Western blotting. The upper panels show a representative experiment and the graph shows HIF-1α protein expression normalized to β-actin. ***p<0.005 *versus* control; ^###^ *p*<0.005 *versus* lactate alone; *n* = 3–8. (B) As in (A) but with WiDr TCs. *ns*, *p*>0.05 *versus* control; *n* = 3–8. (C–F) TCs were infected with a control shRNA (shCTR, left panels) or with a specific shRNA targeting MCT1 (shMCT1-1, right panels). The cells were then cultured during 24-h in the presence of 10 mM lactate or not (control), after which HIF-1 activity was quantified using a dual reporter luciferase assay. The assay was performed using (C) SiHa (*n* = 5–7), (D) HeLa (*n* = 3–4), (E) FaDu (*n* = 5–8), and (F) WiDr (*n* = 4–5) TCs. *ns*, *p*>0.05, *p<0.05, ** *p*<0.01, ***p<0.005 *versus* control.

To test the contribution of MCT1 to lactate signaling in normoxic TCs, we first used α-cyano-4-hydroxycinnamate (CHC), a drug known to reversibly inhibit MCT1 with ∼10-fold selectivity *versus* other MCTs [41]. Used at a concentration of 5 mM, CHC was previously shown not to induce SiHa or WiDr cell death in the presence of glucose [26]. This concentration of the drug totally blocked lactate-induced HIF-1α protein expression in SiHa TCs (Figure 5A), thus confirming that lactate uptake is an upstream event mandatory for triggering this signaling pathway in oxidative TCs and further stressing out an important contribution of MCT1 in this process. Conversely, CHC did not modulate HIF-1α expression in glycolytic WiDr TCs exposed to lactate (Figure 5B), and the same experiment confirmed that these cells were insensitive to HIF-1α protein stabilization by exogenous lactate, as also shown in Figure 1F. Of note, CHC in the presence of glucose but no lactate (i.e., our control conditions) did not modify basal HIF-1α expression in SiHa and WiDr TCs (Figure S5).

We then used a RNA interference approach to better define the role of MCT1 in lactate signaling. TCs were infected with a lentivirus carrying a specific shRNA against MCT1 (shMCT1-1) or a control shRNA (shCTR). We verified in SiHa TCs that shMCT1-1 was specific of MCT1 *versus* MCT4 as it decreased *SLC16A1*/MCT1 but not *SLC16A3*/MCT4 mRNA expression (Figure S2B); protein target extinction in all the cell lines investigated is shown in Figure S2C. In oxidative TCs, MCT1 silencing resulted in a complete loss of HIF-1 activation by lactate, whereas cells infected with shCTR kept full sensitivity; the levels of HIF-1 activation by lactate in shCTR SiHa (Figure 5C), shCTR HeLa (Figure 5D) and shCTR FaDu (Figure 5E) TCs were similar to those reported for wild-type cells in Figure 3A. The contribution of MCT1 to lactate signaling was verified with a second anti-MCT1 shRNA (shMCT1-2) in normoxic SiHa TCs: shMCT1-2 fully inhibited lactate-induced HIF-1α protein stabilization in the cells (Figure S4B) despite a significant increase in *SLC16A3*/MCT4 transcription (Figure S2B). Altogether, these data demonstrate the absolute requirement of MCT1 over other MCTs to activate HIF-1 with lactate in normoxic oxidative TCs. We further found that lactate did not activate HIF-1 in WiDr glycolytic TCs expressing MCT1 (shCTR) or not (shMCT1) (Figure 5F and Figure S2C). As observed in wild-type cells (see Figure 1F), lactate did not induce HIF-1α protein expression in shCTR-infected WiDr TCs (Figure S6A). These last sets of data suggest that Warburg-phenotype TCs are resistant to lactate-induced HIF-1 activation under normoxia, which was confirmed using a second well-known Warburg-phenotype TC line [42]: similar to WiDr, HCT116 human colon carcinoma TCs did not activate HIF-1 in response to lactate (Figure S6B).

### Targeting MCT1 in tumor cells inhibits lactate-induced tumor growth and angiogenesis

**Figure 6:**
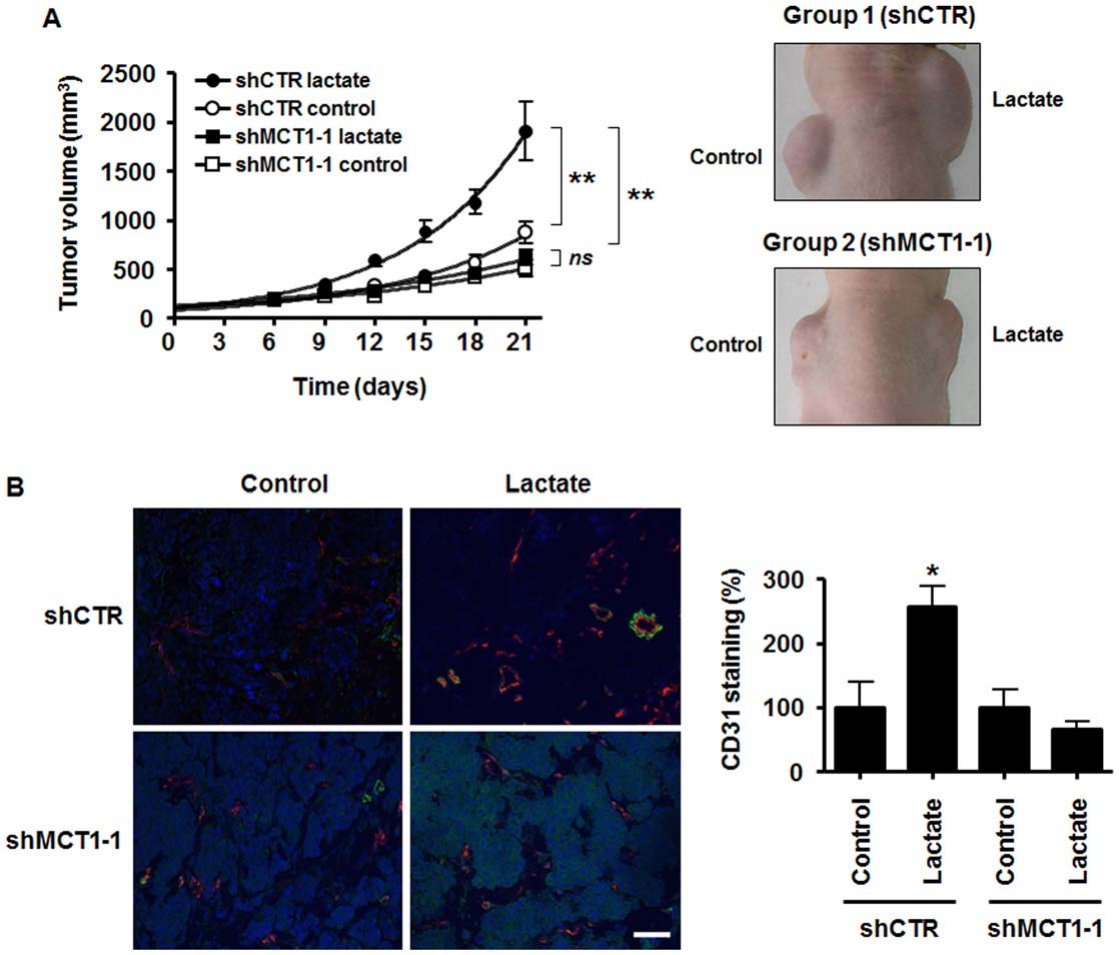
MCT1 expression in tumor cells regulates lactate-induced angiogenesis and tumor growth. Two groups of BALB/c nude mice were injected s.c. with Matrigel plugs containing 30 mM lactate (right flank) or and equal volume of saline (left flank). The plugs also contained 10^6^ SiHa TCs infected with a control shRNA (shCTR, Group 1) or 10^6^ SiHa TCs infected with a specific shRNA against MCT1 (shMCT1-1, Group 2). (A) Tumor growth was tracked over time. The graph shows tumor growth curves and the pictures are representative of mice 21 days after tumor implantation. **p<0.01; *ns*, *p*>0.05; *n* = 5 for Group 1; *n* = 4 for Group 2. (B) In a second set of experiments, plugs were microdissected 12 days after implantation. Pictures show CD31-positive endothelial cells (red) and α-smooth muscle actin-positive pericytes (green). Nuclei are stained in blue with DAPI. Bar = 50 µm. CD31 staining was quantified and is expressed as % of positive surface area in the graph. *n* = 3–4 for Group 1, *n* = 4–5 for Group 2. *p<0.05.

The ability of exogenous lactate to activate HIF-1 in oxidative TCs requires the lactate transporter MCT1 (see Figure 5), which could offer a new therapeutic perspective for the use of MCT1 inhibitors. Since VEGF can be induced by lactate in several cell types [37] including TCs (Figure 3C and reference [18]), we finally addressed *in vivo* whether targeting MCT1 expression in TCs could disrupt tumor angiogenesis. To do so, we developed an *in vivo* model in which lactate was delivered from a growth factor-reduced Matrigel plug to SiHa TCs expressing a specific shRNA against MCT1 (shMCT1-1) or a control shRNA (shCTR). Lactate was used as a sodium salt and did not *per se* modify extracellular pH in our experimental conditions (before injection, pH with and without 30 mM lactate was 7.490±0.006 and 7.487±0.003, respectively, *n* = 3). On Day 0, a first group of mice simultaneously received shCTR SiHa TCs in a lactate-containing plug (right flank) and shCTR SiHa TCs in a lactate-free plug (left flank). In this model, lactate very significantly promoted the growth of otherwise slow growing human tumor xenografts in nude mice (Figure 6A, *p*<0.01, *n* = 5). Growth stimulation by lactate was already evident 12-days after implantation, with a mean tumor volume of 594±62 mm^3^ in the lactate plugs overcoming that of 327±53 mm^3^ in the control plugs (*p* = 0.0114, *n* = 5). On Day +21, mean tumor volumes were of 1907±298 mm^3^ and 874±112 mm^3^ in the lactate and control plugs, respectively (*p* = 0.0118, *n* = 5). A second group of littermate animals underwent the same experimental protocol except that shMCT1-1 SiHa TCs were used. Target extinction is shown in Figure S2C. In this model, MCT1 deletion in the TCs resulted in a full loss of the tumor growth-promoting activity of lactate (Figure 6A). There was no difference in the growth rate of shMCT1-1 SiHa TCs in the presence or in the absence of exogenous lactate (*p*>0.05, *n* = 4). Even more striking was the dramatic inhibition of tumor growth in the presence of lactate when the TCs lacked MCT1 (Figure 6A, *p*<0.01 when comparing shCTR lactate *versus* shMCT1-1 lactate, *n* = 4–5). On Day +12, in the presence of lactate, mean tumor volumes reached 594±62 mm^3^ when TCs expressed MCT1 and only 283±47 mm^3^ in the absence of MCT1 (*p* = 0.0062, *n* = 4–5). On Day +21, the respective tumor volumes were 1907±298 mm^3^ with MCT1 and 616±80 mm^3^ without MCT1 (*p* = 0.0073, *n* = 4–5). The persistence of MCT1 extinction was confirmed at the end of the treatment using immunohistochemistry with plug biopsies (Figure S7).

The contribution of MCT1 expression in TCs to tumor angiogenesis was evidenced in a second set of experiments using the same protocol except that mice were sacrificed and plugs collected for immunohistochemistry on Day +12. Tumor growth curves are shown in Figure S8. Endothelial cell (CD31) staining revealed that lactate compared to saline increased the tumor vascular content, but only when TCs expressed MCT1 (Figure 6B). Pericytes were stained for α-smooth muscle actin. The staining showed a better vascular coverage in the presence of lactate compared to saline, which was lost when TCs did not express MCT1 (Figure 6B).

## Discussion

Tumors are heterogeneous metabolic entities in which malignant cell subpopulations perform OXPHOS, anaerobic glycolysis or aerobic glycolysis. Maintenance of a high rate of glycolysis is under the positive control of HIF-1, a transcription factor associated with cancer aggressiveness and poor prognosis [43], [44]. In this study, we report the reverse phenomenon of a glycolytic control of HIF-1 in TCs. This control is paracrine with lactate known to be produced by glycolytic tumor cells triggering a normoxic activation of HIF-1 in oxidative but not in Warburg phenotype TCs. It was verified using different TC lines with a well-established dependency either on OXPHOS (SiHa, HeLa, FaDu) or on aerobic glycolysis (WiDr, HCT116) [26], [28], [29], [42], and confirmed with isogenic oxidative wild-type and glycolytic ρ0 SiHa TCs. Our findings could impact cancer therapy. We indeed identified the lactate transporter MCT1 as a mandatory upstream component of lactate signaling in oxidative TCs and report that MCT1 expression in TCs is critical for lactate-induced tumor angiogenesis and tumor growth *in vivo*, providing a new rationale for the development and clinical evaluation of MCT1 inhibitors in anticancer therapy.

Lactate is not a metabolic dead-end product but rather a metabolic fuel for oxidative cells [37] and a signaling molecule in several cell types [21], [37]. In tumors, lactate is at the core of a metabolic symbiosis wherein glycolytic/hypoxic TCs produce lactate from glucose and oxidative/oxygenated TCs use lactate as an oxidative fuel [26]. Stromal cells are an additional source of lactate [45], [46]. Besides its role as a fuel, lactate was proposed to inhibit PHD2 in normoxic TCs, an activity that would involve lactate oxidation to pyruvate and a competition between pyruvate and 2-oxoglutarate [18], [19]. The existence of such signaling is still debated. Indeed, using soft ionization mass spectrometry in *in vitro* nondenaturing conditions, Hewitson *et al.* [23] failed to detect a direct interaction between pyruvate and PHD2 and PHD2 inhibition by pyruvate, thus making of lactate signaling through PHD2 an open question for investigation. Another layer of complexity comes from discrepancies in the level of lactate-induced HIF-1α protein stabilization measured in intact TCs, suggesting that TCs are not equal with respect to lactate signaling. Our study proposes a solution for both paradigms: if indeed lactate competes with 2-oxoglutarate to inhibit PHD2 activity and to trigger HIF-1α expression in normoxic TCs (Figure 2), lactate-induced HIF-1 activation is restricted to those TCs performing OXPHOS to the exclusion of Warburg-phenotype TCs. It was confirmed not only in an isogenic TC model but also in different wild-type TC lines sourced from different types of human cancers. It is of note that the different sensitivity to lactate that we detected among different cell lines reflected the level of maximal HIF-1 induction by hypoxia. Experimental variability was also found in a same oxidative cell line at different passages (compare Figure 1E and Figure S1A). This could be due, at least in part, to the intrinsic metabolic profiles of the cells: most TCs have an intermediate metabolism contributed by both OXPHOS and glycolysis [47], [48]. If pyruvate instead of directly interacting with the 2-oxoglutarate-binding pocket of PHD2 rather indirectly interferes with the binding of 2-oxoglutarate, it would explain why Hewitson and colleagues using *N*-terminally truncated PHD2 failed to evidence enzyme inhibition by pyruvate [23]. Our study and other reports [18], [19], [21], [22] in intact malignant and nonmalignant cells expressing full length PHD2 provide collective evidence that PHD2 inhibition accounts for HIF-1 activation by lactate and pyruvate. This response was lost upon PHD2 targeting with a specific siRNA (Figure 2C).

When aerobically activated, HIF-1 has also been suggested to contribute to the Warburg phenotype [10], [24]. However, we rather evidenced resistance to HIF-1α expression in Warburg-phenotype TCs. It was first observed when comparing basal protein levels in oxidative SiHa *versus* glycolytic WiDr TCs (Figure 1B) and oxidative wild-type SiHa *versus* glycolytic ρ0 SiHa TCs (Figure 3B). In addition, we did not find evidence of a glycolytic control of basal HIF-1α expression in WiDr Warburg-phenotype TCs through autocrine lactate signaling (Figure 5F & Figure S6). The molecular mechanisms coupling the glycolytic switch to HIF-1 repression certainly warrants further investigation. They could involve changes in intracellular pyruvate, 2-oxoglutarate and/or reactive oxygen species availability.

A paracrine control of HIF-1 by lactate offers new therapeutic perspectives for cancer. As a master regulator of both glycolytic and angiogenic switches, HIF-1 has been proposed as a target for therapy [11]. But although several drugs have been identified to exert anticancer effects partially through HIF-1 inhibition [49], there is currently no small drug inhibitor reported to directly target HIF-1. In this regard, an important finding is the necessary involvement of MCT1 in lactate-induced HIF-1 activation in oxidative TCs (Figure 5). Lactate shuttles contribute in many ways to cancer growth, lactate being used as an oxidative fuel [26], [34] or as a signaling agent in several cell types [37]. Our study further evidenced VEGF induction by lactate in oxidative but not in Warburg-phenotype TCs (Figure 3C–E), which not only clarifies the scope of previous findings [18] but also supports the stimulation by lactate of paracrine VEGF signaling. In this context, it is important to stress out that hypoxia mimicry by lactate extends to nonmalignant cells. In normoxic endothelial cells, we indeed recently showed that lactate triggers HIF-1 activity through supporting a competition between pyruvate and 2-oxoglutarate for PHD2 [22] similar to what we observed in TCs. However, final effectors differ. If on the one hand basic fibroblast growth factor (bFGF) rather than VEGF was found to be the main pro-angiogenic effector of lactate signaling in endothelial cells (lactate did not increase endothelial VEGF production), lactate further induced the expression of VEGFR2, the main transducer of pro-angiogenic VEGF signaling, in endothelial cells. With respect to HIF-1 activation, the pro-angiogenic activity of lactate thus comprises the stimulation of paracrine VEGF signaling through acting on both VEGF-producing TCs (this study) and VEGFR2-expressing target endothelial cells [22], and the stimulation of autocrine bFGF signaling in endothelial cells [22]. In endothelial cells, PHD2 inhibition is further involved in the induction of nuclear factor-κB by lactate, which supports autocrine pro-angiogenic interleukin-8 signaling [21]. The importance of lactate signaling in TCs was underscored in this study by the *in vivo* observation that the tumor growth-promoting and pro-angiogenic activities of lactate depend on MCT1 expression in TCs (Figure 6). Enhanced vascular pericyte coverage in lactate-treated tumors suggests that lactate may further stimulate HIF-2, a PHD-regulated transcription factor involved in vascular maturation [50].

The antitumor effects of MCT1 deficiency in TCs that we evidenced *in vivo* (Figure 6) provide a new rationale for the therapeutic use of MCT1 inhibitors in cancer. Basal HIF-1 expression was not sensitive to MCT1 inhibition in the cell lines analyzed (Figure S5) and lactate flux inhibition was previously reported not to induce cell death in the presence of glucose [26], providing overt clinical safety to the strategy. Warburg-phenotype TCs were, however, resistant to lactate signaling. Because MCT1 activity is driven by the proton gradient across the plasma membrane [32], this observation could be explained by the net outward flux of lactic acid coupled to high-rate glycolysis (Figure 1A), together with moderate expression of MCT1 (Figure 4A) and of its anchor protein CD147 (Figure 4B). Given the glycolytic activity of Warburg TCs, the higher expression of LDH-H detected in WiDr *versus* oxidative SiHa TCs (Figure 4C) could reflect increased expression of heterotetrameric intermediate LDH isoforms preferentially catalyzing the reduction of pyruvate into lactate.

To conclude, our study unraveled a metabolic control of HIF-1 accounting for paracrine lactate-induced HIF-1 activation in normoxic oxidative TCs, whereas Warburg-phenotype TCs are intrinsically resistant. MCT1 is revealed as a key upstream component of lactate signaling in oxidative TCs, thus extending the therapeutic repertoire of MCT1 inhibitors to direct anti-angiogenic effects on TCs.

## Materials and Methods

### Cells and reagents

All cell lines were from ATCC. SiHa human cervix squamous cell carcinoma, WiDr human colorectal adenocarcinoma, FaDu human pharynx squamous cell carcinoma, HeLa human epithelial cervix cancer, and HCT116 human colorectal carcinoma cancer cells were cultured in DMEM containing *D*-glucose (4,500 mg/l), *L*-glutamine (4 mM), heat-inactivated FBS (10%) and 1% penicillin-streptomycin. Mitochondrial DNA-depleted (ρ0) SiHa cells were produced and cultured as described previously [51]. Glucose-deprived DMEM was from Krackeler Scientific and was used without FBS, glutamine and antibiotics. All experiments (including with ρ0 cells) were performed in pyruvate-free media buffered at pH 7.3 (3.7 g/l NaHCO_3_, 5% CO_2_). To further avoid extracellular pH effects, lactate was used as a sodium salt. Hypoxia (1% O_2_) was achieved using a hypoxia workstation (Ruskinn). To minimize artifactual variations due to unequal growth rates and cell sizes, all assays were performed on confluent cells in fresh medium. Cell death was quantified using the NucleoCounter device from ChemoMetec. Unless stated otherwise, all drugs were from Sigma.

### Western blotting and immunostaining

Western blotting was performed as previously shown [52], except that lysates were not heat-denaturated for HIF-1α protein detection. This allowed the detection of basal HIF-1α that would otherwise not be detected [53]. For MCT1 and CD147 immunostaining, cells were permeabilized with 0.1% triton X-100 in PBS. We used previously disclosed protocols for bright-light immunohistochemistry [54] and immunofluorescence [26]. Hoechst 33342 or 4′,6-diamidino-2-phenylindole (DAPI) were used to stain cell nuclei and Mayer's hematoxylin for counterstaining where indicated. *In situ* protein interaction was tested using the proximity ligation assay Duolink kit from Olink Bioscience according to manufacturer's instructions. Briefly, the assay detects protein interactions through using a pair of secondary antibodies coupled to a positive (probe PLUS) and a negative (probe MINUS) DNA strand. DNA-coupled antibody binding is followed by *in situ* DNA hybridization, amplification and detection steps with a fluorescently labeled probe (Duolink detection kit 563). Primary antibodies were: a mouse monoclonal against HIF-1α (BD), a rabbit polyclonal against PHD2 (Novus Biologicals), a rabbit polyclonal against MCT1 (Millipore), a mouse monoclonal against CD147/basigin (BD), a mouse monoclonal against LDH-H (Novus Biologicals), a mouse monoclonal against β-actin (Sigma), a rat monoclonal against CD31 (BD), and a rabbit polyclonal against α-smooth muscle actin (Abcam). Areas of positive CD31 staining in mouse tumor cryosections were quantified using the Framework for Image Dataset Analysis (FRIDA) software developed at Johns Hopkins University.

### Lactate measurements

Lactate concentration was measured in the deproteinized supernatant of confluent cells using the enzymatic assay commercialized by CMA Microdialysis AB on a CMA600 analyzer (Aurora Borealis).

### Determination of PHD2 and HIF-1 activities

Dual luciferase reporter assays were performed with the dual luciferase kit (DLR) from Promega using pODD-Luc and pGL3-(PGK-HRE6)-TK-Luc as reporters of PHD [17], [22] and HIF-1 [16] activities, respectively. The reporter plasmid and a Renilla transfection normalization vector (Promega) were admixed (10∶1) before dilution (1.6%) in OptiMEM (Invitrogen). Cells were transfected with TransIT-2020 transfection reagent (Mirus) for PHD activity assays or with Lipofectamine 2000 (Invitrogen) for the determination of HIF-1 activity.

### Quantitative RT-PCR and RNA interference

Reverse transcription was performed from total RNA using RevertAid M-MuLV Reverse Transcriptase and random hexamer primers (Fermentas). The following primers were used for SYBR green quantitative PCR on a Biorad IQ5 system [55]. hVEGF-A sense, 5′-TACCTCCACCATGCCAAGTG-3′, antisense, 5′-ATGATTCTGCCCTCCTCCTTC-3′; hHIF-1α sense, 5′-TCATCCATGTGACCATGAGG-3′, antisense, 5′-TTCTTCCTCGGCTAGTTAGGG-3′; hMCT1/SLC16A1 sense, 5′-GTGGCTCAGCTCCGTATTGT-3′, antisense, 5′-GAGCCGACCTAAAAGTGGTG-3′; hMCT2/SLC16A7 sense, 5′-CAACACCATTCCAAGACAGC-3′, antisense, 5′-TGGCTGTTATGTACGCAGGA-3′; hMCT3/SLC16A8 sense, 5′-GGATGTGTTGAAGAACTATGAGATC-3′, antisense 5′-CCGGGTTCCTCTGCAACA-3′; hMCT4/SLC16A3 sense, 5′-CAGTTCGAGGTGCTCATG G-3′, antisense, 3′-ATGTAGACGTGGGTCGCATC-5′; hCD147 sense, 5′-TGCTGGTCTGCAAGTCAGAG-3′, antisense, 5′-GCGAGGAACTCACGAAGAAC-3′; hLDH-B sense, 5′-CACCAGTTGCGGAAGAAGA-3′, antisense, 5′-CCAAAACATCCACAAGAGCA-3′; and housekeeping human ribosomal protein L19 (hRPL19) sense, 5′-CAAGCGGATTCTCATGGAACA-3′, antisense, 5′-TGGTCAGCCAGGAGCTTCTT-3′. For siRNA experiments, SiHa TCs were transfected with a specific siRNA against PHD2 (siPHD2, 5′-GACGAAAGCCAUGGUUGCUUG[dTdT]-3′, Eurofins) [15] using the lipofectamine RNAiMAX reagent (Invitrogen) according manufacturer's protocol. Allstar siRNA (Qiagen) was used as a negative control (siCTR). For shRNA experiments, cells were infected with a lentiviral vector containing the selected shRNA, as previously disclosed [56]. MCT1 shRNAs were purchased from Open Biosystems (clone TRCN0000038340 = shMCT1-1 and clone TRCN0000038478 = shMCT1-2). Control shRNA (shCTR) was Addgene plasmid 1864.

### *In vivo* experiments

All *in vivo* experiments were performed with approval of the *Université catholique de Louvain* (UCL) authorities (*Comité d'Ethique Facultaire pour l'Expérimentation Animale*, specific approval ID for this study was TUMETABO) according to national animal care regulations. Eight week-old male BALB/c nude mice were purchased from Elevage Janvier and randomly assigned to a treatment group. Anesthetized (ketamine/xylazine) mice in Group 1 were injected subcutaneously with two plugs of 300-µl growth-factor-reduced Matrigel: the plug in the right flank contained 10^6^ SiHa TCs infected with a control shRNA and 30 mM sodium *L*-lactate, and the plug in the left flank contained 10^6^ SiHa TCs infected with a control shRNA and lactate was replaced by an equal volume of saline. Anesthetized (ketamine/xylazine) mice in Group 2 underwent the same treatment protocol except that SiHa TCs were infected with a specific shRNA against MCT1 (shMCT1-1). To avoid extracellular pH effects, lactate was used as a sodium salt. In a first set of experiments, tumor growth was determined every 3 days during 21 days. Longest and shortest diameters were measured with an electronic caliper and the formula of a prolate ellipsoid was used to calculate the volume of the tumor [57], [58]. In a second set of experiments, mice injected with a lethal dose of ketamine/xylazine were sacrificed by cervical dislocation 12 days after Matrigel plug implantation. The plugs were microdissected, snap-frozen in liquid nitrogen-cooled isopentane, and used for immunostaining as described above.

### Statistical analyses

Results are expressed as mean ± SEM. In some figures, error bars are smaller than symbols. Student's *t* test, 1-way ANOVA (Tukey's post-hoc test) or 2-way ANOVA were used where appropriate. *P*<0.05 was considered to be statistically significant.

## Supporting Information

Figure S1 **Lactate stabilizes HIF-1α posttranscriptionally in normoxic SiHa tumor cells.** (A) HIF-1α and β-actin protein expression was detected using Western blotting in the lysates of SiHa TCs treated during 24-h with 10 mM lactate, 10 mM of pyruvate or not. The upper panels show representative experiments and the graphs HIF-1α protein expression normalized to β-actin levels. *p<0.05; *n* = 6–9. (B) *HIF-1α* mRNA expression was detected using RT-qPCR in normoxic SiHa TCs treated with 10 mM lactate during the indicated amounts of time. Data are normalized to *RPL19* mRNA expression. *n* = 3–6.
(TIF)

Figure S2 **Target extinction after siRNA/shRNA delivery.** (A) PHD2 and β-actin were detected in SiHa TC lysates using Western blotting 48-h after transfection with a specific siRNA against PHD2 (siPHD2) or a control siRNA (siCTR). (B) *SLC16A1*/MCT1 (left graph) and *SLC16A3*/MCT4 (right graph) mRNA expression was detected using RT-qPCR in SiHa TCs expressing a control shRNA (shCTR) or a shRNA against MCT1 (shMCT1-1 = Open Biosystems clone TRCN0000038340, shMCT1-2 = Open Biosystems clone TRCN0000038478). *ns*, *p*>0.05, **p<0.01 *versus* shCTR; *n* = 4–9. (C) MCT1 and β-actin were detected using Western blotting in the different TC lines used in this study. The cells were infected with a lentivirus carrying a control shRNA (shCTR) or a specific shRNA against MCT1 (shMCT1-1). Representative Western Blots are shown. For SiHa TCs, MCT1 and β-actin expression are shown at 4th and 11th *in vitro* passages after shRNA delivery to emphasize the long-term persistence of MCT1 extinction.
(TIF)

Figure S3 **Hypoxic activation of HIF-1 in SiHa and HeLa tumor cells.** SiHa (*n* = 3) and HeLa (*n* = 3–4) TCs in fresh medium were cultured during 24-h under normoxia or hypoxia (1% O_2_). HIF-1 activity was quantified using a dual reporter luciferase assay. ***p<0.005.
(TIF)

Figure S4 **MCT1 gates lactate-induced HIF-1α protein stabilization in normoxic SiHa tumor cells.** (A) The relative expression of mRNAs encoding MCT1 to 4 was determined using RT-qPCR in untreated SiHa TCs. *n* = 3. (B) SiHa TCs were infected with a control shRNA (shCTR) or with a specific shRNA targeting MCT1 (shMCT1-2). The cells were then cultured during 24-h in the presence of 10 mM lactate or not (control), after which HIF-1α and β-actin expression was detected using Western blotting. The upper panels show representative experiments and the graphs HIF-1α protein expression normalized to β-actin levels. *ns*, *p*>0.05, *p<0.05, ***p<0.005; *n* = 4–6.
(TIF)

Figure S5 **MCT1 inhibition by CHC does not modify basal HIF-1α expression in tumor cells.** Confluent SiHa and WiDr TCs in fresh medium were treated during 24-h with 5 mM of α-cyano-4-hydroxycinnamate (CHC) or not. HIF-1α and β-actin were detected using Western blotting. The panels show representative blots.
(TIF)

Figure S6 **Lactate does not activate HIF-1 in Warburg-phenotype tumor cells.** (A–B) TCs were cultured during 24-h in fresh medium containing 10 mM lactate or not (control). (A) WiDr TCs were expressing a control shRNA (shCTR). HIF-1α and β-actin were detected using Western blotting. The upper panels show a representative blot and the graph shows HIF-1α protein expression normalized to β-actin. *ns*, *p*>0.05; *n* = 4. (B) HIF-1 activity was quantified using a dual reporter luciferase assay in HCT116 human colorectal carcinoma cells. *ns*, *p*>0.05; *n* = 3–4.
(TIF)

Figure S7 **Long-term extinction of MCT1** ***in vivo*** **.** Mice on Day +21 of the experiment shown in Figure 6A were sacrificed and the tumor plugs of the lactate treatment conditions were microdissected. Typical pictures show MCT1 staining in cryosections of the plugs that contained SiHa TCs infected with a control shRNA (shCTR) or with a specific shRNA against MCT1 (shMCT1-1) at the time of plug implantation on Day 0. Bar = 10 µm.
(TIF)

Figure S8 **Growth curves of SiHa tumor plugs in nude mice.** Two groups of BALB/c nude mice were injected s.c. with Matrigel plugs containing 30 mM lactate (right flank) or and equal volume of saline (left flank). The plugs also contained 10^6^ SiHa TCs infected with a control shRNA (shCTR, *n* = 4) or 10^6^ SiHa TCs infected with a specific shRNA against MCT1 (shMCT1-1, *n* = 5). Tumor growth was tracked over time and is shown in the graph. All mice were sacrificed on Day +12 for the immunohistochemical determination of angiogenesis in plug biopsies shown in Figure 6B.
(TIF)

## References

[1] Hanahan D, Weinberg RA (2011) Hallmarks of cancer: the next generation. Cell 144: 646–674. 21376230 10.1016/j.cell.2011.02.013

[2] Porporato PE, Dadhich RK, Dhup S, Copetti T, Sonveaux P (2011) Anticancer targets in the glycolytic metabolism of tumors: a comprehensive review. Front Pharmacol 2: 49. 21904528 10.3389/fphar.2011.00049 PMC3161244

[3] Wu R, Racker E (1959) Regulatory mechanisms in carbohydrate metabolism. IV. Pasteur effect and Crabtree effect in ascites tumor cells. J Biol Chem 234: 1036–1041. 13654314

[4] Warburg O, Wind F, Negelein E (1927) The metabolism of tumors in the body. J Gen Physiol 8: 519–530. 19872213 10.1085/jgp.8.6.519 PMC2140820

[5] Pollard PJ, Wortham NC, Tomlinson IP (2003) The TCA cycle and tumorigenesis: the examples of fumarate hydratase and succinate dehydrogenase. Ann Med 35: 632–639. 14708972 10.1080/07853890310018458

[6] Selak MA, Armour SM, MacKenzie ED, Boulahbel H, Watson DG, et al (2005) Succinate links TCA cycle dysfunction to oncogenesis by inhibiting HIF-alpha prolyl hydroxylase. Cancer Cell 7: 77–85. 15652751 10.1016/j.ccr.2004.11.022

[7] Dang L, White DW, Gross S, Bennett BD, Bittinger MA, et al (2009) Cancer-associated IDH1 mutations produce 2-hydroxyglutarate. Nature 462: 739–744. 19935646 10.1038/nature08617 PMC2818760

[8] Fantin VR, St Pierre J, Leder P (2006) Attenuation of LDH-A expression uncovers a link between glycolysis, mitochondrial physiology, and tumor maintenance. Cancer Cell 9: 425–434. 16766262 10.1016/j.ccr.2006.04.023

[9] Moreno-Sanchez R, Rodriguez-Enriquez S, Marin-Hernandez A, Saavedra E (2007) Energy metabolism in tumor cells. FEBS J 274: 1393–1418. 17302740 10.1111/j.1742-4658.2007.05686.x

[10] Luo W, Hu H, Chang R, Zhong J, Knabel M, et al (2011) Pyruvate Kinase M2 Is a PHD3-Stimulated Coactivator for Hypoxia-Inducible Factor 1. Cell 145: 732–744. 21620138 10.1016/j.cell.2011.03.054 PMC3130564

[11] Semenza GL (2003) Targeting HIF-1 for cancer therapy. Nat Rev Cancer 3: 721–732. 13130303 10.1038/nrc1187

[12] Semenza GL (2010) HIF-1: upstream and downstream of cancer metabolism. Curr Opin Genet Dev 20: 51–56. 19942427 10.1016/j.gde.2009.10.009 PMC2822127

[13] Pugh CW, Ratcliffe PJ (2003) Regulation of angiogenesis by hypoxia: role of the HIF system. Nat Med 9: 677–684. 12778166 10.1038/nm0603-677

[14] Hirsila M, Koivunen P, Gunzler V, Kivirikko KI, Myllyharju J (2003) Characterization of the human prolyl 4-hydroxylases that modify the hypoxia-inducible factor. J Biol Chem 278: 30772–30780. 12788921 10.1074/jbc.M304982200

[15] Berra E, Benizri E, Ginouves A, Volmat V, Roux D, et al (2003) HIF prolyl-hydroxylase 2 is the key oxygen sensor setting low steady-state levels of HIF-1alpha in normoxia. EMBO J 22: 4082–4090. 12912907 10.1093/emboj/cdg392 PMC175782

[16] Maxwell PH, Wiesener MS, Chang GW, Clifford SC, Vaux EC, et al (1999) The tumour suppressor protein VHL targets hypoxia-inducible factors for oxygen-dependent proteolysis. Nature 399: 271–275. 10353251 10.1038/20459

[17] Li F, Sonveaux P, Rabbani ZN, Liu S, Yan B, et al (2007) Regulation of HIF-1alpha stability through S-nitrosylation. Mol Cell 26: 63–74. 17434127 10.1016/j.molcel.2007.02.024 PMC2905600

[18] Lu H, Forbes RA, Verma A (2002) Hypoxia-inducible factor 1 activation by aerobic glycolysis implicates the Warburg effect in carcinogenesis. J Biol Chem 277: 23111–23115. 11943784 10.1074/jbc.M202487200

[19] Lu H, Dalgard CL, Mohyeldin A, McFate T, Tait AS, et al (2005) Reversible inactivation of HIF-1 prolyl hydroxylases allows cell metabolism to control basal HIF-1. J Biol Chem 280: 41928–41939. 16223732 10.1074/jbc.M508718200

[20] Elvidge GP, Glenny L, Appelhoff RJ, Ratcliffe PJ, Ragoussis J, et al (2006) Concordant regulation of gene expression by hypoxia and 2-oxoglutarate-dependent dioxygenase inhibition: the role of HIF-1alpha, HIF-2alpha, and other pathways. J Biol Chem 281: 15215–15226. 16565084 10.1074/jbc.M511408200

[21] Vegran F, Boidot R, Michiels C, Sonveaux P, Feron O (2011) Lactate influx through the endothelial cell monocarboxylate transporter MCT1 supports an NF-kappaB/IL-8 pathway that drives tumor angiogenesis. Cancer Res 71: 2550–2560. 21300765 10.1158/0008-5472.CAN-10-2828

[22] Sonveaux P, Copetti T, De Saedeleer CJ, Vegran F, Verrax J, et al (2012) Targeting the lactate transporter MCT1 in endothelial cells inhibits lactate-induced HIF-1 activation and tumor angiogenesis. PLoS ONE 7: e33418. 22428047 10.1371/journal.pone.0033418 PMC3302812

[23] Hewitson KS, Lienard BM, McDonough MA, Clifton IJ, Butler D, et al (2007) Structural and mechanistic studies on the inhibition of the hypoxia-inducible transcription factor hydroxylases by tricarboxylic acid cycle intermediates. J Biol Chem 282: 3293–3301. 17135241 10.1074/jbc.M608337200

[24] Semenza GL (2007) HIF-1 mediates the Warburg effect in clear cell renal carcinoma. J Bioenerg Biomembr 39: 231–234. 17551816 10.1007/s10863-007-9081-2

[25] Walenta S, Schroeder T, Mueller-Klieser W (2004) Lactate in solid malignant tumors: potential basis of a metabolic classification in clinical oncology. Curr Med Chem 11: 2195–2204. 15279558 10.2174/0929867043364711

[26] Sonveaux P, Vegran F, Schroeder T, Wergin MC, Verrax J, et al (2008) Targeting lactate-fueled respiration selectively kills hypoxic tumor cells in mice. J Clin Invest 118: 3930–3942. 19033663 10.1172/JCI36843 PMC2582933

[27] Walenta S, Snyder S, Haroon ZA, Braun RD, Amin K, et al (2001) Tissue gradients of energy metabolites mirror oxygen tension gradients in a rat mammary carcinoma model. Int J Radiat Oncol Biol Phys 51: 840–848. 11699496 10.1016/s0360-3016(01)01700-x

[28] Reitzer LJ, Wice BM, Kennell D (1979) Evidence that glutamine, not sugar, is the major energy source for cultured HeLa cells. J Biol Chem 254: 2669–2676. 429309

[29] Rideout D, Bustamante A, Patel J (1994) Mechanism of inhibition of FaDu hypopharyngeal carcinoma cell growth by tetraphenylphosphonium chloride. Int J Cancer 57: 247–253. 8157363 10.1002/ijc.2910570220

[30] Forsythe JA, Jiang BH, Iyer NV, Agani F, Leung SW, et al (1996) Activation of vascular endothelial growth factor gene transcription by hypoxia-inducible factor 1. Mol Cell Biol 16: 4604–4613. 8756616 10.1128/mcb.16.9.4604 PMC231459

[31] Kong D, Park EJ, Stephen AG, Calvani M, Cardellina JH, et al (2005) Echinomycin, a small-molecule inhibitor of hypoxia-inducible factor-1 DNA-binding activity. Cancer Res 65: 9047–9055. 16204079 10.1158/0008-5472.CAN-05-1235

[32] Halestrap AP, Price NT (1999) The proton-linked monocarboxylate transporter (MCT) family: structure, function and regulation. Biochem J 343 Pt 2: 281–299. 10510291 PMC1220552

[33] Halestrap AP, Meredith D (2004) The SLC16 gene family-from monocarboxylate transporters (MCTs) to aromatic amino acid transporters and beyond. Pflugers Arch 447: 619–628. 12739169 10.1007/s00424-003-1067-2

[34] Whitaker-Menezes D, Martinez-Outschoorn UE, Lin Z, Ertel A, Flomenberg N, et al (2011) Evidence for a stromal-epithelial “lactate shuttle” in human tumors: MCT4 is a marker of oxidative stress in cancer-associated fibroblasts. Cell Cycle 10: 1772–1783. 21558814 10.4161/cc.10.11.15659 PMC3142461

[35] Ullah MS, Davies AJ, Halestrap AP (2006) The plasma membrane lactate transporter MCT4, but not MCT1, is up-regulated by hypoxia through a HIF-1alpha-dependent mechanism. J Biol Chem 281: 9030–9037. 16452478 10.1074/jbc.M511397200

[36] Dimmer KS, Friedrich B, Lang F, Deitmer JW, Broer S (2000) The low-affinity monocarboxylate transporter MCT4 is adapted to the export of lactate in highly glycolytic cells. Biochem J 350 Pt 1: 219–227. 10926847 PMC1221245

[37] Dhup S, Dadhich RK, Porporato PE, Sonveaux P (2012) Multiple biological activities of lactic acid in cancer: influences on tumor growth, angiogenesis and metastasis. Curr Pharm Des 18: 1319–1330. 22360558 10.2174/138161212799504902

[38] Wilson MC, Meredith D, Fox JE, Manoharan C, Davies AJ, et al (2005) Basigin (CD147) is the target for organomercurial inhibition of monocarboxylate transporter isoforms 1 and 4: the ancillary protein for the insensitive MCT2 is EMBIGIN (gp70). J Biol Chem 280: 27213–27221. 15917240 10.1074/jbc.M411950200

[39] Finch NA, Linser PJ, Ochrietor JD (2009) Hydrophobic interactions stabilize the basigin-MCT1 complex. Protein J 28: 362–368. 19760495 10.1007/s10930-009-9202-3

[40] Fredriksson S, Gullberg M, Jarvius J, Olsson C, Pietras K, et al (2002) Protein detection using proximity-dependent DNA ligation assays. Nat Biotechnol 20: 473–477. 11981560 10.1038/nbt0502-473

[41] Manning Fox JE, Meredith D, Halestrap AP (2000) Characterisation of human monocarboxylate transporter 4 substantiates its role in lactic acid efflux from skeletal muscle. J Physiol 529 Pt 2: 285–293. 11101640 10.1111/j.1469-7793.2000.00285.x PMC2270204

[42] Weinberg F, Hamanaka R, Wheaton WW, Weinberg S, Joseph J, et al (2010) Mitochondrial metabolism and ROS generation are essential for Kras-mediated tumorigenicity. Proc Natl Acad Sci U S A 107: 8788–8793. 20421486 10.1073/pnas.1003428107 PMC2889315

[43] Unruh A, Ressel A, Mohamed HG, Johnson RS, Nadrowitz R, et al (2003) The hypoxia-inducible factor-1 alpha is a negative factor for tumor therapy. Oncogene 22: 3213–3220. 12761491 10.1038/sj.onc.1206385

[44] Brahimi-Horn MC, Chiche J, Pouyssegur J (2007) Hypoxia and cancer. J Mol Med 85: 1301–1307. 18026916 10.1007/s00109-007-0281-3

[45] Martinez-Outschoorn UE, Pavlides S, Howell A, Pestell RG, Tanowitz HB, et al (2011) Stromal-epithelial metabolic coupling in cancer: Integrating autophagy and metabolism in the tumor microenvironment. Int J Biochem Cell Biol 43: 1045–1051. 21300172 10.1016/j.biocel.2011.01.023 PMC3102770

[46] Fiaschi T, Marini A, Giannoni E, Taddei ML, Gandellini P, et al (2012) Reciprocal metabolic reprogramming through lactate shuttle corrdinately influences tumor-stroma interplay. Cancer Res 10.1158/0008-5472.CAN-12-1949 [doi]. 10.1158/0008-5472.CAN-12-1949 22850421

[47] Gatenby RA, Gillies RJ (2004) Why do cancers have high aerobic glycolysis? Nat Rev Cancer 4: 891–899. 15516961 10.1038/nrc1478

[48] Vander Heiden MG, Cantley LC, Thompson CB (2009) Understanding the Warburg effect: the metabolic requirements of cell proliferation. Science 324: 1029–1033. 19460998 10.1126/science.1160809 PMC2849637

[49] Onnis B, Rapisarda A, Melillo G (2009) Development of HIF-1 inhibitors for cancer therapy. J Cell Mol Med 13: 2780–2786. 19674190 10.1111/j.1582-4934.2009.00876.x PMC2832082

[50] Semenza GL (2009) Involvement of oxygen-sensing pathways in physiologic and pathologic erythropoiesis. Blood 114: 2015–2019. 19494350 10.1182/blood-2009-05-189985

[51] King MP, Attardi G (1989) Human cells lacking mtDNA: repopulation with exogenous mitochondria by complementation. Science 246: 500–503. 2814477 10.1126/science.2814477

[52] Feron O, Belhassen L, Kobzik L, Smith TW, Kelly RA, et al (1996) Endothelial nitric oxide synthase targeting to caveolae. Specific interactions with caveolin isoforms in cardiac myocytes and endothelial cells. J Biol Chem 271: 22810–22814. 8798458 10.1074/jbc.271.37.22810

[53] Quintero M, Colombo SL, Godfrey A, Moncada S (2006) Mitochondria as signaling organelles in the vascular endothelium. Proc Natl Acad Sci U S A 103: 5379–5384. 16565215 10.1073/pnas.0601026103 PMC1459363

[54] Sonveaux P, Brouet A, Havaux X, Gregoire V, Dessy C, et al (2003) Irradiation-induced angiogenesis through the up-regulation of the nitric oxide pathway: implications for tumor radiotherapy. Cancer Res 63: 1012–1019. 12615716

[55] Bouzin C, Brouet A, De Vriese J, DeWever J, Feron O (2007) Effects of vascular endothelial growth factor on the lymphocyte-endothelium interactions: identification of caveolin-1 and nitric oxide as control points of endothelial cell anergy. J Immunol 178: 1505–1511. 17237399 10.4049/jimmunol.178.3.1505

[56] Moon EJ, Sonveaux P, Porporato PE, Danhier P, Gallez B, et al (2010) NADPH oxidase-mediated reactive oxygen species production activates hypoxia-inducible factor-1 (HIF-1) via the ERK pathway after hyperthermia treatment. Proc Natl Acad Sci U S A 107: 20477–20482. 21059928 10.1073/pnas.1006646107 PMC2996638

[57] Stragand JJ, Barlogie B, White RA, Drewinko B (1981) Biological properties of the human colonic adenocarcinoma cell line SW 620 grown as a xenograft in the athymic mouse. Cancer Res 41: 3364–3369. 7260902

[58] Tomayko MM, Reynolds CP (1989) Determination of subcutaneous tumor size in athymic (nude) mice. Cancer Chemother Pharmacol 24: 148–154. 2544306 10.1007/BF00300234

