# Supplementary figures and images for: Lactate Activates HIF-1 in Oxidative but Not in Warburg-Phenotype Human Tumor Cells
Source: PLoS One. 2012 Oct 17;7(10):e46571. doi: 10.1371/journal.pone.0046571 (PMC3474765; doi:10.1371/journal.pone.0046571)

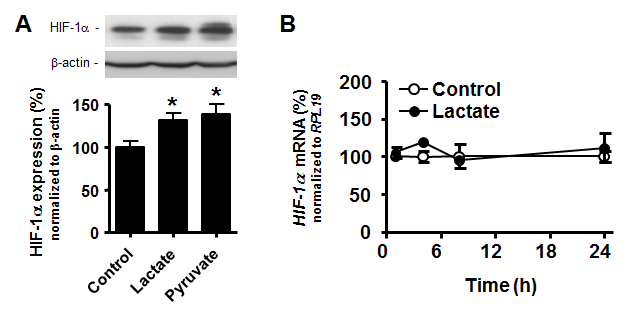

Supplement: Figure S1 — Lactate stabilizes HIF-1α posttranscriptionally in normoxic SiHa tumor cells. (A) HIF-1α and β-actin protein expression was detected using Western blotting in the lysates of SiHa TCs treated during 24-h with 10 mM lactate, 10 mM of pyruvate or not. The upper panels show representative experiments and the graphs HIF-1α protein expression normalized to β-actin levels. *p<0.05; n = 6–9. (B) HIF-1α mRNA expression was detected using RT-qPCR in normoxic SiHa TCs treated with 10 mM lactate during the indicated amounts of time. Data are normalized to RPL19 mRNA expression. n = 3–6. (TIF) [file pone.0046571.s001.tif]

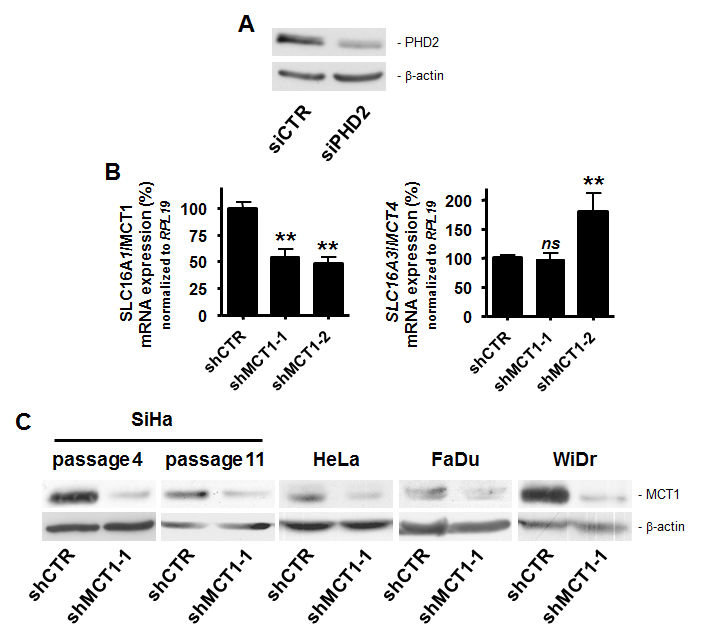

Supplement: Figure S2 — Target extinction after siRNA/shRNA delivery. (A) PHD2 and β-actin were detected in SiHa TC lysates using Western blotting 48-h after transfection with a specific siRNA against PHD2 (siPHD2) or a control siRNA (siCTR). (B) SLC16A1/MCT1 (left graph) and SLC16A3/MCT4 (right graph) mRNA expression was detected using RT-qPCR in SiHa TCs expressing a control shRNA (shCTR) or a shRNA against MCT1 (shMCT1-1 = Open Biosystems clone TRCN0000038340, shMCT1-2 = Open Biosystems clone TRCN0000038478). ns, p>0.05, **p<0.01 versus shCTR; n = 4–9. (C) MCT1 and β-actin were detected using Western blotting in the different TC lines used in this study. The cells were infected with a lentivirus carrying a control shRNA (shCTR) or a specific shRNA against MCT1 (shMCT1-1). Representative Western Blots are shown. For SiHa TCs, MCT1 and β-actin expression are shown at 4th and 11th in vitro passages after shRNA delivery to emphasize the long-term persistence of MCT1 extinction. (TIF) [file pone.0046571.s002.tif]

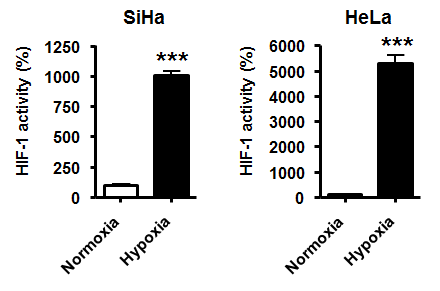

Supplement: Figure S3 — Hypoxic activation of HIF-1 in SiHa and HeLa tumor cells. SiHa (n = 3) and HeLa (n = 3–4) TCs in fresh medium were cultured during 24-h under normoxia or hypoxia (1% O2). HIF-1 activity was quantified using a dual reporter luciferase assay. ***p<0.005. (TIF) [file pone.0046571.s003.tif]

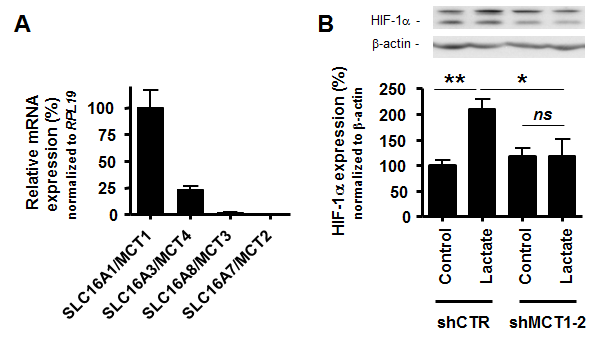

Supplement: Figure S4 — MCT1 gates lactate-induced HIF-1α protein stabilization in normoxic SiHa tumor cells. (A) The relative expression of mRNAs encoding MCT1 to 4 was determined using RT-qPCR in untreated SiHa TCs. n = 3. (B) SiHa TCs were infected with a control shRNA (shCTR) or with a specific shRNA targeting MCT1 (shMCT1-2). The cells were then cultured during 24-h in the presence of 10 mM lactate or not (control), after which HIF-1α and β-actin expression was detected using Western blotting. The upper panels show representative experiments and the graphs HIF-1α protein expression normalized to β-actin levels. ns, p>0.05, *p<0.05, ***p<0.005; n = 4–6. (TIF) [file pone.0046571.s004.tif]

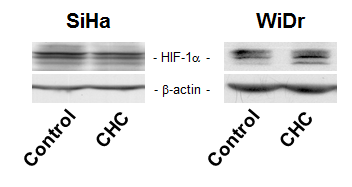

Supplement: Figure S5 — MCT1 inhibition by CHC does not modify basal HIF-1α expression in tumor cells. Confluent SiHa and WiDr TCs in fresh medium were treated during 24-h with 5 mM of α-cyano-4-hydroxycinnamate (CHC) or not. HIF-1α and β-actin were detected using Western blotting. The panels show representative blots. (TIF) [file pone.0046571.s005.tif]

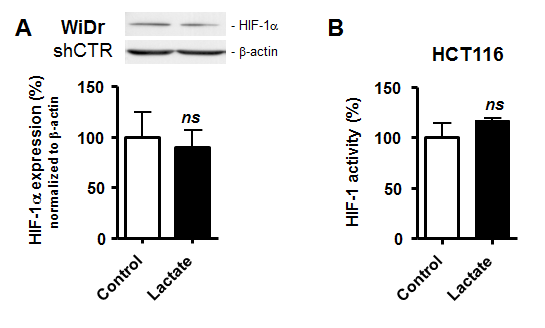

Supplement: Figure S6 — Lactate does not activate HIF-1 in Warburg-phenotype tumor cells. (A–B) TCs were cultured during 24-h in fresh medium containing 10 mM lactate or not (control). (A) WiDr TCs were expressing a control shRNA (shCTR). HIF-1α and β-actin were detected using Western blotting. The upper panels show a representative blot and the graph shows HIF-1α protein expression normalized to β-actin. ns, p>0.05; n = 4. (B) HIF-1 activity was quantified using a dual reporter luciferase assay in HCT116 human colorectal carcinoma cells. ns, p>0.05; n = 3–4. (TIF) [file pone.0046571.s006.tif]

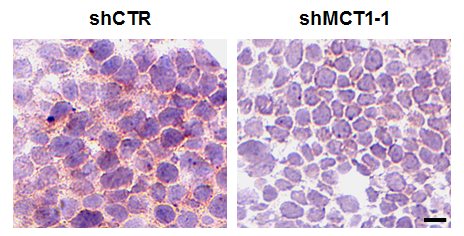

Supplement: Figure S7 — Long-term extinction of MCT1 in vivo . Mice on Day +21 of the experiment shown in Figure 6A were sacrificed and the tumor plugs of the lactate treatment conditions were microdissected. Typical pictures show MCT1 staining in cryosections of the plugs that contained SiHa TCs infected with a control shRNA (shCTR) or with a specific shRNA against MCT1 (shMCT1-1) at the time of plug implantation on Day 0. Bar = 10 µm. (TIF) [file pone.0046571.s007.tif]

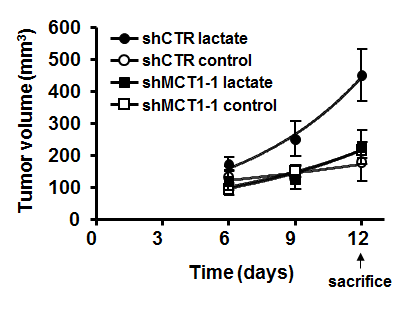

Supplement: Figure S8 — Growth curves of SiHa tumor plugs in nude mice. Two groups of BALB/c nude mice were injected s.c. with Matrigel plugs containing 30 mM lactate (right flank) or and equal volume of saline (left flank). The plugs also contained 106 SiHa TCs infected with a control shRNA (shCTR, n = 4) or 106 SiHa TCs infected with a specific shRNA against MCT1 (shMCT1-1, n = 5). Tumor growth was tracked over time and is shown in the graph. All mice were sacrificed on Day +12 for the immunohistochemical determination of angiogenesis in plug biopsies shown in Figure 6B. (TIF) [file pone.0046571.s008.tif]
